# Overexpression of Outer Membrane Protein X (OmpX) Compensates for the Effect of TolC Inactivation on Biofilm Formation and Curli Production in Extraintestinal Pathogenic *Escherichia coli* (ExPEC)

**DOI:** 10.3389/fcimb.2018.00208

**Published:** 2018-06-22

**Authors:** Binyou Li, Qi Huang, Ailian Cui, Xueling Liu, Bo Hou, Liyuan Zhang, Mei Liu, Xianrong Meng, Shaowen Li

**Affiliations:** Key Laboratory of Preventive Veterinary Medicine in Hubei Province, College of Veterinary Medicine, Huazhong Agricultural University, Wuhan, China

**Keywords:** extraintestinal pathogenic *Escherichia coli*, outer membrane protein X, TolC, biofilm formation, curli production

## Abstract

Our previous study showed that the inactivation of the efflux pump TolC could abolish biofilm formation and curli production of extraintestinal pathogenic *Escherichia coli* (ExPEC) strain PPECC42 under hyper-osmotic conditions. In this study we investigated the role of OmpX in biofilm formation and curli production of ExPEC PPECC42. Our data showed that OmpX disruption or overexpression didn't significantly affect the biofilm formation and curli production of the wild-type strain. However, in the *tolC*-deleted mutant, overexpressing OmpX suppressed the effect of TolC inactivation on ExPEC biofilm formation and curli production under hyper-osmotic growth conditions. Real-time qRT-PCR confirmed that OmpX overexpression affected curli production by regulating the transcription of the curli biosynthesis-related genes in the Δ*tolC* strain. Our findings suggest that OmpX is involved in biofilm formation and curli production.

## Introduction

Extraintestinal pathogenic *Escherichia coli* (ExPEC) are a sub group of pathogenic *E. coli* strains causing a variety of infections and diseases at extraintestinal sites in humans and animals, which are typically characterized by multi-organ infections including urinary tract infections, meningitis, polyserositis, and septicemia (Johnson and Russo, 2005; Smith et al., 2007; Köhler and Dobrindt, 2011; Manges and Johnson, 2012; Mitchell et al., 2015). Recently, ExPEC strains were frequently isolated from clinical samples of pigs (Ding et al., 2012). Moreover, they were widely found in pork, retail chicken, beef, and ready-to-eat foods, which pose a potential threat to public health (Lyhs et al., 2012; Aslam et al., 2014; Mitchell et al., 2015).

Biofilms are defined as structured communities of bacterial cells enclosed in a self-produced polymeric matrix and adhered to an inert or living surface (Costerton et al., 1999). Biofilms can increase bacterial resistance to external environmental stresses such as exposure to antimicrobials, antiseptics, desiccation, and extremes of temperature, which can help the bacteria to survive in different hostile environments (Steenackers et al., 2012; de La Fuente-Núñez et al., 2013). Biofilms can also serve as a physical barrier to protect bacteria from eradication by the host immune defense system (Donlan and Costerton, 2002), and are considered to be an important virulence factor in ExPEC (Magistro et al., 2015). Therefore, exploration of the role of biofilm formation in pathogenesis and virulence is important in investigating factors that influence biofilm formation and for addressing means to prevent or inhibit/eradicate biofilms in the production and human settings.

Biofilm formation is a complicated process and controlled by complex networks in *E. coli*. Bacterial active motility achieved by flagella movement increases the chance of bacteria to interact with surfaces (Donlan, 2002). Once attached to the surface, bacterial fimbriae, mainly type 1 fimbriae and curli, and adhesins, such as Ag43, promote adhesion to surfaces (Danese et al., 2000; Holden and Gally, 2004). After initial attachment, bacteria produce a number of extracellular components constituting the biofilm matrix, mainly composed of amyloid curli fibers and cellulose. Biofilm formation involves considerable regulations at transcriptional as well as post-transcriptional levels. Environmental changes affecting biofilm formation are sensed by several two-component systems, including CpxA/R, RcsC/D/B, and EnvZ/OmpR, which mediate transcriptional regulations of genes involving outer membrane protein production, flagellar synthesis, curli expression etc. (Prigent-Combaret et al., 2001; Otto and Silhavy, 2002; Ferrières and Clarke, 2003). The global transcription factors, including RpoS, H-NS, and BolA, and the master regulators for curli fimbriae expression, CsgD, are believed to play dominant roles in the transcriptional regulation of biofilm formation (reviewed in Mika and Hengge, 2014). Small molecules, including the well-known second messenger c-di-GMP, the alarmone ppGpp, N-acetylglucosamine-6-P (GlcNAc-6P), and the auto-inducer-2 (AI-2), function as post-transcriptional factors regulating bacterial biofilm formation. (Balzer and Mclean, 2002; Ren et al., 2004; Barnhart et al., 2006; González Barrios et al., 2006; Jenal and Malone, 2006).

Curli fimbriae play important roles in the irreversible adhesion stage of *E. coli* biofilm formation to form self-produced extracellular matrix, enhance initial cell-cell interactions, and adhesion to biotic and abiotic surfaces, and eventually promote biofilm formation (Austin et al., 1998; White et al., 2003; Barnhart and Chapman, 2006; Beloin et al., 2008). Curli biosynthesis-related genes in *E. coli* cluster in two divergent operons: *csgDEFG* and *csgBAC* (Van Houdt and Michiels, 2005). The *csgBAC* operon encodes the major structural subunit CsgA and the core protein CsgB (Hammar et al., 1995, 1996). The *csgDEFG* operon encodes four accessory proteins which facilitate translocation of the curli subunits across the outer membrane and contribute to curli assembly and stability, in which CsgD is an essential positive transcriptional regulator of the curli regulatory network. The expression of curli biosynthesis-related genes is also regulated by several other transcription factors, such as RpoS and H-NS (Austin et al., 1998; Chapman et al., 2002).

Our previous study showed that inactivation of TolC compromised the ability of biofilm formation and curli production in porcine ExPEC strain PPECC42 in response to hyper-osmotic conditions (Hou et al., 2014). TolC is the major channel for drug efflux across the outer membrane of *E. coli*, and is well-known for its involvement in the transportation of various types of chemicals, including antibiotics, disinfectants, and metabolic products, leading to bacterial antimicrobial resistance (Zgurskaya et al., 2011). Other than a drug efflux pump, TolC has also been reported to be involved in biofilm formation in *Actinobacillus pleuropneumoniae*, a porcine respiratory tract pathogen (Li et al., 2016). However, the underlying mechanism of how TolC affects biofilm formation remains largely unknown. In order to explain how TolC affected biofilm formation, we carried out a comparative proteomic analysis which showed that the expression of outer membrane protein X (OmpX) was abolished in the Δ*tolC* strain detected by using SDS-PAGE combined with MALDI-TOF mass-spectrometry (data not shown).

OmpX and its homologs have been identified in many Gram-negative bacteria, such as *Enterobacter cloacae* (OmpX) (Stoorvogel et al., 1991)*, Salmonella enterica* serovar Typhimurium (PagC, Rck) (Heffernan et al., 1992), *Yersinia* spp. (Ail) (Kolodziejek et al., 2010), *E. coli* (OmpX, Lom) (Mecsas et al., 1995), and *Klebsiella pneumoniae* (OmpK17) (Climent et al., 1997). These proteins are of low molecular weight (from 15 to 18 kDa) and fold in an eight-β-barrel structure with membrane-spanning domains that protrude from the cell surface. They are involved in considerable number of physiological processes including binding external proteins, participating in channeling, antibiotic resistance, signal transduction, invasion, survival in macrophages, internalization in epithelial cells and virulence (Stoorvogel et al., 1991; Vogt and Schulz, 1999; Miller et al., 2001; Otto and Hermansson, 2004; Kolodziejek et al., 2010; Meng et al., 2016). OmpX overproduction was considered to be a bacterial adaptive response toward environmental stresses (Dupont et al., 2007). However, the role of OmpX in bacterial biofilm formation remains unclear. The present study investigated the role of OmpX in ExPEC biofilm formation using both gene deletion and overexpression assays. Our data showed that OmpX overexpression suppressed the effect of *tolC* inactivation on ExPEC biofilm formation and curli production in response to the hyper-osmotic stress.

## Materials and methods

### Strain construction

Strains, plasmids, and primers used in this work are listed in Table 1. The wild-type (WT) ExPEC strain PPECC42 was isolated from the lung of a diseased pig in China in 2006. Its complete genome sequence has been submitted to NCBI (Genbank Accession No. NZ_CM003707.1). An isogenic *omp*X-deleted strain of ExPEC PPECC42, in which a 345 bp fragment was deleted within the *ompX* open reading frame (ORF), was constructed as described previously (Meng et al., 2016). To construct the strains overexpressing OmpX, the plasmid pHSG*::ompX* containing the full-length *ompX* gene of ExPEC strain PPECC42 (Meng et al., 2016) was electroporated into each strain needed. Clones were selected on LB agar plates containing chloramphenicol. The primers used in this work are shown in Table 1. All strains were grown either in Lysogeny broth (LB) or in M9 or 1/2 M9 minimal medium. When necessary, ampicillin or chloramphenicol was used at 100 and 25 μg/ml, respectively.

**Table 1:** Strains, plasmids, and primers used in this work.

	**Description or sequence**	**Source**
**STRAIN**		
ExPEC strain PPECC42	Wild-type (WT), porcine origin, Cm^S^	Hou et al., 2014
Δ*tolC*	Mutant with a 158-bp fragment deleted from the whole ORF of the *tolC* gene in PPECC42, Cm^S^	Hou et al., 2014
Δ*ompX*	Mutant with a 516-bp fragment deleted from the whole ORF of the *ompX* gene in PPECC42, Cm^S^	Meng et al., 2016
Δ*tolC::ompX*	Δ*tolC* mutant carrying plasmid *pHSG::ompX*, Cm^R^	This study
*WT::ompX*	PPECC42 strain carrying plasmid *pHSG::ompX*, Cm^R^	This study
χ7213	Thi-1 thr-1 leuB6 fhuA21 lacY1 glnV44ΔasdA4 recA1 RP4 2-Tc::Mu[λpir] KmR	Hou et al., 2014
DH5α	F–,ϕ80dlacZΔM15, Δ(lacZYA-argF) U169,deoR, recA1, endA1, hsdR17 (rk^−^,mk^+^), phoA,supE44, λ^−^,thi-1,gyrA96,relA1	Takara Bio
**PLASMID**		
pRE112	oriT oriVΔasdCm^R^SacB, suicide vector	Hou et al., 2014
pHSG	ori lacZ Cm^R^ *pHSG396*	Takara Bio
pHSG::*ompX*	ori lacZ Cm^R^ *pHSG396* with the full-length *ompX* gene	Meng et al., 2016
**PRIMER**		
*ompX_*F*_*	ACCTGAAATACCGCTATGAA	
*ompX_*R*_*	TCAGTGGTCTGGAATTTACC	
*csgD_*F*_*	CCCGTACCGCGACATTG	
*csgD_*R*_*	ACGTTCTTGATCCTCCATGGA	
*csgB_*F*_*	CATAATTGGTCAAGCTGGGACTAA	
*csgB_*R*_*	GCAACAACCGCCAAAAGTTT	
*GAPDH_*F*_*	ACTTACGAGCAGATCAAAGC	
*GAPDH_*R*_*	AGTTTCACGAAGTTGTCGTT	

### Determination of growth kinetics

Fresh LB medium, M9 or 1/2 M9 minimal medium was inoculated with a 1:100 dilution of overnight cultures and incubated at 37°C under shaking at 200 *rpm*. Samples were collected every hour and the optical densities were measured at 600 nm (OD_600_) using a BioPhotometer (Eppendorf, Hamburg, Germany). The data were obtained from three independent experiments, with each having three biological replicates.

### Crystal violet biofilm assay

Biofilm formation was evaluated using crystal violet assay as described previously (Stepanovic et al., 2000). Briefly, the overnight grown bacterial culture was diluted 1:100 in each indicated medium in a 96-well microtiter plate (Nunc, Denmark) and incubated at 28°C. The wells without bacterial inoculation were taken as the negative control. After 120 h, the cells were removed, and the wells were washed five times with sterile distilled water. The wells were stained with 125 μl of 1.0% (w/v) crystal violet for 15 min, washed with sterile distilled water, and the biofilm was then dissolved with 150 μl of 33% (v/v) glacial acetic acid. The optical density of each well was measured at 630 nm (OD_630_) using a Universal Microplate Reader (Bio-Tek, Winooski, USA). All assays were performed with 12 replicates. The cutoff OD (ODc) was defined as three times standard deviation above the mean OD of the negative control.

### Visualization of curli fimbriae

Curli production was assessed by morphological examination of colonies grown on M9 or 1/2 M9 agar plates containing 40 μg/mL Congo red (Amresco, Ohio, USA) and 20 μg/mL Coomassie brilliant blue (Solarbio, Beijing, China) as described previously (Lloyd et al., 2012). Briefly, 1 μL of the overnight grown culture of each strain was spotted onto the Congo red plate and incubated at 28°C for 120 h. The morphology of each bacterial spot was observed and imaged. The experiments were carried out in triplicate.

### Real-time quantitative reverse transcription polymerase chain reaction (qRT-PCR)

Total RNAs were extracted using the RNeasy Mini Kit (OMEGA, Minnesota, USA) from the cells of each strain cultured in different conditions. cDNA was reverse transcribed from 2 μg of RNA using HiScript TM first strand cDNA synthesis kit (Vazyme, New Jersey, USA), and used as the template for qRT-PCR with AceQ™ qPCR SYBR Green Master Mix Kit (Vazyme, New Jersey, USA) using a Bio-Rad detection system (Bio-Rad, California, USA). The expression of the target genes in each strain was normalized to that of the house-keeping gene *GAPDH* using the delta-delta threshold cycle (ΔΔCT) method (Viveiros et al., 2007), with mutants Ct values representing the fold change relative to that of the WT strain, which was set at 1. Comparative qRT-PCR was used to determine the average expression from four replicate wells. The assays were repeated using RNAs harvested from independent cultures of each strain in triplicate.

### Statistical analysis

Statistical analyses were performed using GraphPad prism version 5.0 software (GraphPadPrism Software, San Diego, CA). Statistical difference was calculated using the One-way ANOVA test and considered significant at a *p* < 0.05.

## Results

### Disruption or overexpression of OmpX does not affect cell growth

**Figure 1:**
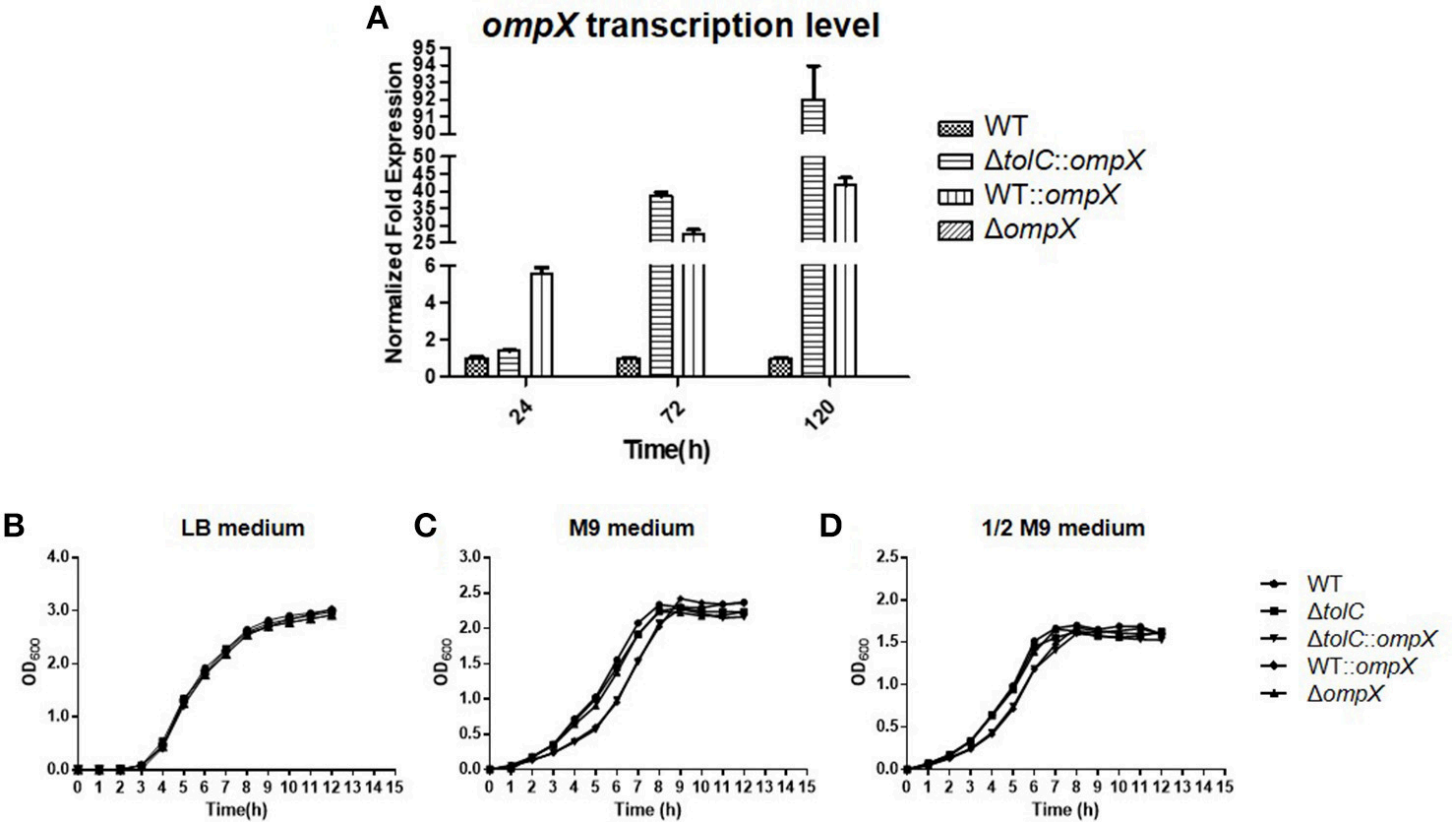
Deleting or overexpressing *ompX* alone does not affect the cell growth. **(A)** Verification of ompX deletion and overexpression. Cells of each indicated strain were grown in the M9 medium at 28°C and collected at each indicated time point. mRNA was extracted from each sample and the expression level of *ompX* was determined by using real-time qRT-PCR. **(B–D)** Growth curves. Cells of each indicated strain were inoculated from overnight grown culture into LB **(B)**, M9 medium **(C)**, and 1/2 M9 medium **(D)**, and grown at 37°C. OD_600_ was measured every hour. The data shown are the mean and standard deviation of three independent cultures.

OmpX overexpression was achieved by introducing plasmid pHSG*::ompX* into each strain. The expression level of OmpX in each strain was analyzed by using real-time qRT-PCR. As shown in Figure 1A, at each indicated time-point (24, 72, and 120 h) of growth, expression of OmpX was not detected in the Δ*ompX* mutant, but was detected in the Δ*tolC::ompX* and WT*::ompX* strains, suggesting that the strains construction was successful. The growth curves of each strain were determined in LB medium, M9 minimal medium, and 1/2 M9 minimal medium, and the results showed that all the strains presented a similar growth pattern to the WT strain (Figures 1B–D), indicating that OmpX inactivation and overexpression do not significantly affect the growth of ExPEC.

### Deletion or overexpression of OmpX does not significantly affect the biofilm formation of the wild-type expec

**Figure 2:**
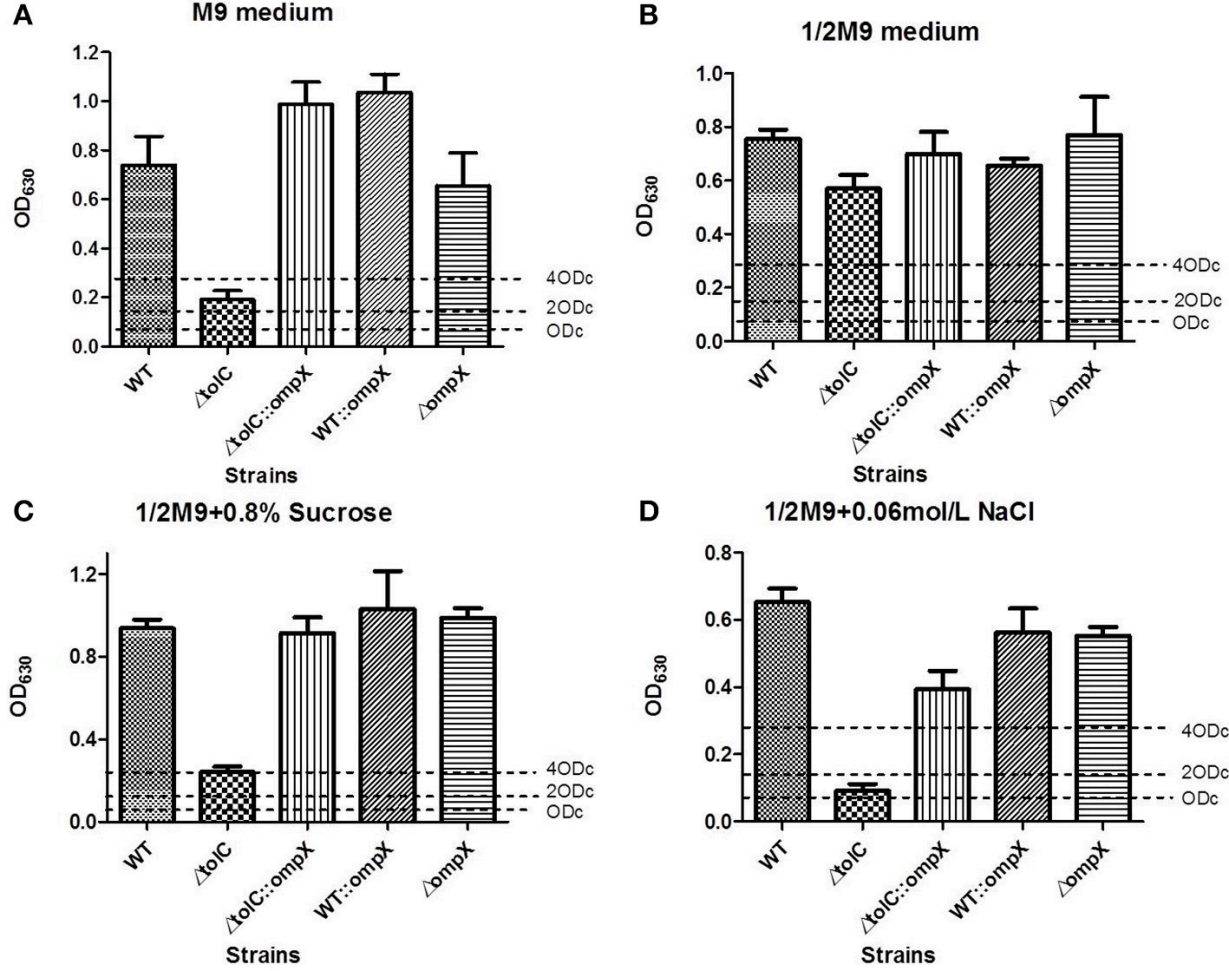
Biofilm formation of the Δ*tolC* strain under hyper-osmotic conditions was restored by overproducing OmpX. Cells of each indicated strain were inoculated from overnight grown culture into 1/2 M9 **(A)**, M9 **(B)**, 1/2M9 supplemented with 0.8% sucrose **(C)**, and 1/2 M9 supplemented with 0.06 M NaCl **(D)**. The cells were collected after 120 h growth at 28°C and the biofilms were measured using crystal violet biofilm assay. The data shown are the mean of 12 biological replicates. The cutoff OD (ODc) was defined as three times standard deviation above the mean OD_630_ value of the negative control.

We next investigated the effect of OmpX deletion and overexpression on biofilm formation of ExPEC. Cells were grown in 1/2 M9 or M9 medium at 28°C for 120 h and the biofilm formation was measured using crystal violet biofilm assay. As shown in Figures 2A,B, the wild-type strain with overexpressed OmpX (indicated as WT::*ompX*) or deleted *ompX* (indicated as *ompX*) showed a comparable level of biofilm formation to the wild-type strain (WT), indicating the deletion or overexpression of OmpX alone does not affect the biofilm formation of the wild-type ExPEC.

### Overexpression of OmpX restored the biofilm formation of the Δ*tolC* strain under hyper-osmotic conditions

Our previous study revealed that the disruption of *tolC* led to decreased biofilm formation when the cells were grown in M9 medium (Hou et al., 2014). We next tested whether OmpX has a similar role on the *tolC* deletion strain and influencing its ability to form biofilms. As shown in Figure 2A, the wild-type strain showed a strong ability of biofilm formation while the Δ*tolC* strain almost lost the ability of biofilm formation, which was consistent with our previous study (Hou et al., 2014). In contrast, overexpressing OmpX in the Δ*tolC* mutant (indicated as Δ*tolC*::*ompX*) restored the ability to form biofilm to a similar level of the wild-type strain (Figure 2A), suggesting that the overproduction of OmpX compensated for the defective biofilm formation caused by *tolC* inactivation in the M9 medium. It was seen that in 1/2 M9 medium, all the strains formed strong biofilms (Figure 2B). Considering the difference between M9 and 1/2 M9 media in osmolarity, we speculated that the influence of OmpX overexpression on biofilm formation might be related to external osmotic response. Therefore, the biofilm formation of the Δ*tolC* strain with or without overexpressed OmpX was measured in sucrose- and NaCl- induced hyper-osmotic conditions, respectively. As shown in Figures 2C,D in both conditions, the overexpression of OmpX was able to restore the ability of biofilm formation of the Δ*tolC* strain. The biofilm formation of the Δ*ompX* strain and the wild-type strain overexpressing OmpX was not affected under these conditions. The above results strongly suggest that OmpX overexpression compensates for the defective biofilm formation induced by hyper-osmotic stresses of the Δ*tolC* strain.

### OmpX overexpression compensates for the defective curli production of the Δ*tolC* mutant

**Figure 3:**
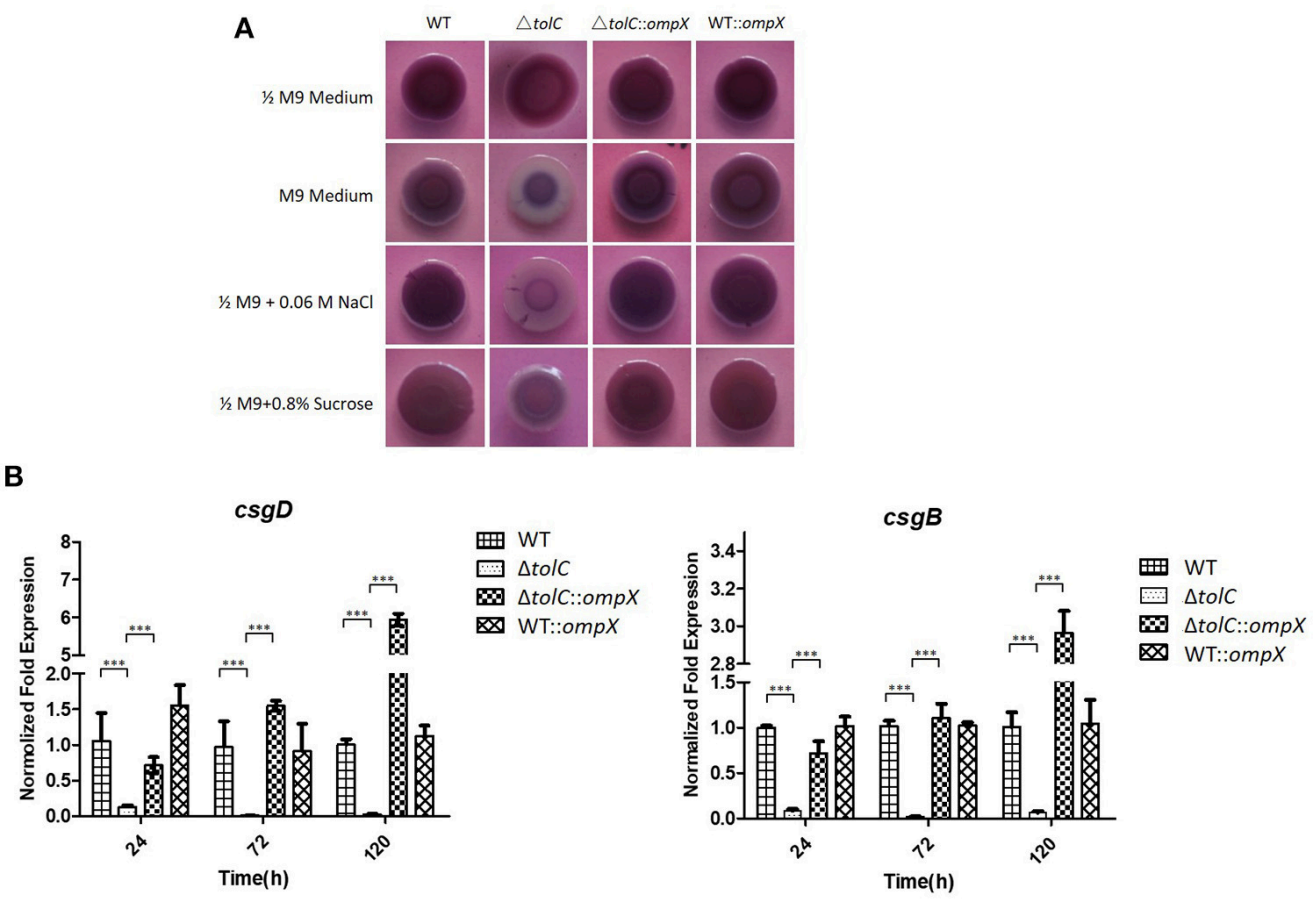
*ompX* overexpression restored the curli production of the Δ*tolC* strain under hyper-osmotic conditions. **(A)** Congo red (CR) assay analysis of curli production. One microliter of overnight grown cells of each indicated stain were spotted to the agar plates containing 40 μg /ml Congo red, which were incubated at 28°C for 120 h. **(B)** Transcription analysis of *csgD* and *csgB*. The cells of each indicated strain were grown in the M9 medium at 28°C and the cells were collected at each indicated timepoint. RNA was extracted and the relative levels were determined using real-time qRT-PCR. The data shown are the mean of three biological replicates. The error bars represented the standard deviations. Significant differences were determined using the One-way Anova test. ^***^P < 0.001.

Our previous study showed that the inactivation of *tolC* reduced the curli production in hyper-osmotic conditions (Hou et al., 2014). So we next investigated whether OmpX overexpression also suppressed the curli production of the Δ*tolC* mutant. A Congo red (CR) assay was used to test the curli production. As shown in Figure 3A, all of the four strains, WT, Δ*tolC*, Δ*tolC*::*ompX*, and WT::*ompX*, presented a *rdar* (red, dry, and rough) morphology on 1/2 M9-CR agar plates, indicating normal curli production. However, in M9 medium and under 0.06 M NaCl- or 0.8% sucrose-induced hyper-osmotic conditions, in contrast with the wild-type strain that still presented a *rdar* morphology, the Δ*tolC* strain showed a significantly whiter morphology, which was consistent with our previous study that the disruption of *tolC* reduced the curli production. Intriguingly, overproducing OmpX restored the morphology of the cells of Δ*tolC* strain to a similar level to the WT strain. This result suggests that OmpX overexpression suppresses the reduced curli production resulted from *tolC* deletion under hyper-osmotic stresses.

The transcription levels of two separate curli biosynthesis-related genes, *csgD* and *csgB*, were further tested in the four strains grown in the M9 medium. As shown in Figure 3B, at each indicated time point of growth, compared with the WT strain, the Δ*tolC* mutant showed a significantly down-regulated expression of both of *csgD* and *csgB* genes. In contrast, the Δ*tolC::ompX* strain exhibited a much higher expression level of sboth genes than the Δ*tolC* strain. The results further suggested that the regulation of OmpX overexpression on curli production is due to its effect on the transcription of the curli biosynthesis-related genes.

## Discussion

Biofilms can increase bacterial resistance to external environmental stresses, and help the bacteria survive in different hostile environments (Steenackers et al., 2012; de La Fuente-Núñez et al., 2013). Efflux pump protein TolC is involved in the transportation of various types of chemicals, including antibiotics, disinfectants, and metabolic products. Our previous study confirmed that inactivation of TolC compromised ExPEC biofilm formation and curli production in response to high osmolarity (Hou et al., 2014). Our preliminary proteomic data showed that the OmpX protein was not detected in the Δ*tolC* mutant, indicating potential interplays between TolC and OmpX. Hence, this study was designed to explore the role of OmpX in ExPEC biofilm formation and curli production, especially under hyper-osmotic conditions, and to determine whether OmpX mediated the effect of TolC inactivation on ExPEC biofilm formation.

Our data showed that the disruption or overexpression of OmpX alone did not have any effect on biofilm formation; however, in the Δ*tolC* background OmpX overexpression significantly suppressed the inhibited biofilm formation and curli production under hyper-osmotic conditions. TolC is able to regulate many proteins and interact with other outer membrane proteins in *E. coli* under different concentrations of glucose (Yang et al., 2011). The deletion of *tolC* increased the transcription of *ompC* and *micF* under high osmotic conditions, whereafter reduced the amount of OmpF (Misra and Reeves, 1987). OmpX overproduction resulted in a decrease in expression of a classical porin Omp36 in *E. aerogenes* (Dupont et al., 2004). Overproduction of OmpX and downregulation of porins were considered to be a bacterial adaptive response toward environmental stresses (Dupont et al., 2007). OmpX overexpression could also increase the activity of σE, an important envelope stress response factor in *E. coli* (Otto and Hermansson, 2004; Pletzer et al., 2015).

Therefore, this study suggests that TolC and OmpX play important roles in responding to and resisting osmotic stresses. When confronting hyper-osmotic conditions, the bacterial cells lacking TolC became unable to carry out proper osmolarity regulations, therefore were sensitive to these stresses. Under this circumstance, OmpX overexpression may modify the expression of NaCl or sucrose transportation related proteins or signal pathways, increase the efflux or decrease the influx of NaCl or sucrose across the outer membrane, and thus relieve the hyper-osmolarity stresses and regulate curli production and biofilm formation. The current study provides a new insight into the role of OmpX in bacterial biofilm formation and environmental osmotic adaptation.

## Author contributions

BL, QH, AC, XL, BH, and LZ performed research. QH, BL, ML, XM, and SL designed research and analyzed the data. BL, QH, and SL wrote the paper.

### Conflict of interest statement

The authors declare that the research was conducted in the absence of any commercial or financial relationships that could be construed as a potential conflict of interest.
